# The Livebirth Rate Per *In Vitro* Fertilization Cycle Is Higher Than The Cumulative Live Birth Rates of Intrauterine Insemination for Patients of Poseidon Group 3 With Unexplained Infertility

**DOI:** 10.3389/fendo.2021.768975

**Published:** 2021-12-01

**Authors:** Yixuan Wu, Haiying Liu, Jianqiao Liu

**Affiliations:** Department of Obstetrics and Gynecology, Center for Reproductive Medicine/Department of Fetal Medicine and Prenatal Diagnosis/BioResource Research Center, Key Laboratory for Major Obstetric Diseases of Guangdong Province, Key Laboratory of Reproductive Medicine of Guangdong Province, The Third Affiliated Hospital of Guangzhou Medical University, Guangzhou, China

**Keywords:** unexplained infertility, poor ovarian reserve, cumulative live birth rate, *in vitro* fertilization, intrauterine insemination

## Abstract

**Background:**

No studies have been done to examine the efficacy of IVF and intrauterine insemination (IUI) for the treatment of young patients with unexplained infertility and low ovarian reserve, although it is becoming an increasingly significant indication for *in-vitro* fertilization (IVF). The goal of this research was to compare the efficacy of IVF with IUI on Poseidon group 3 patients with unexplained infertility (PG3&UI).

**Methods:**

This was a retrospective analysis of PG3&UI patients who had IVF/intracytoplasmic sperm injection (ICSI) or IUI at the Third Affiliated Hospital of Guangzhou Medical University between January 1, 2015, and March 31, 2021. To equalize the baseline characteristics of the IVF/ICSI and IUI groups, propensity score matching (PSM) was utilized. Intention-to-treat (ITT) and per-protocol (PP) analyses were used to compare the differences in live births. To discover variations in time to biochemical pregnancy leading to live birth, Kaplan-Meier curves were produced. To evaluate the expenses per live birth between two procedures, a cost-effective analysis was done.

**Results:**

According to ITT analysis, the live birth rate for the IVF/ICSI group was substantially higher than the cumulative live birth rate (CLBR) for the IUI group (22.6% (38/168) *vs*. 11.3% (19/168), RR 2.00, 95% CI 1.20-3.32, *P* = 0.006). In the PP analysis, the live birth rate was 23.0% (38/165) in the IVF/ICSI group and 11.7% (19/162) in the IUI group (RR = 1.96, 95% CI 1.18-3.26, *P* = 0.007). When censored at 365 days, the Kaplan-Meier analysis revealed that the IVF/ICSI group had a higher live birth rate than the IUI group (log-rank test χ²= 6.025; *P* = 0.014). However, when the two groups were censored at 180 days, the live birth rates were not substantially different (log-rank test χ²= 3.847; *P* = 0.05). The number of hospital visits per live birth in the IUI group was higher than in the IVF/ICSI group (85 *vs*. 48). The overall cost of a live birth was comparable across the two groups (¥132242 *vs*. ¥131611), while the medical expenses for a live delivery from IVF/ICSI were higher than those from IUI (¥118955 *vs*. ¥108279).

**Conclusions:**

The livebirth rate per IVF/ICSI cycle with at most one embryo transfer is higher than the CLBR of IUI, with fewer hospital visits and similar expenses.

## Introduction

Failure to conceive despite a year of regular unprotected intercourse in couples without anovulation, semen abnormalities, tubal disease, or other identifiable infertility reasons is classified as unexplained infertility. It accounts for around 30-40% of causes of infertility (1). IUI and IVF are the most frequent assisted reproductive technologies (ART) used to treat unexplained infertility. Farquhar et al. demonstrated that women who underwent three cycles of IUI with ovarian stimulation (IUI/OS) had a higher CLBR than women who underwent three cycles of expectant management. Women with unexplained infertility should undergo 3 to 4 cycles of IUI with clomiphene or letrozole (1).

Many prior types of research evaluated the efficacy of IVF with IUI or expectant management for unexplained infertility. In a randomized controlled trial (RCT) involving 258 couples, Goverde et al. compared the pregnancy rates of IVF and IUI. They concluded that the IVF group had a greater pregnancy rate each cycle than the IUI group (either IUI alone or IUI/OS). The cumulative pregnancy rate for 6 cycles of IVF, on the other hand, was equivalent to that of IUI (2). Custers et al. compared the effectiveness of a single cycle of IVF with a single embryo transfer against three cycles of IUI/OS. They demonstrated that the two groups had comparable rates of ongoing pregnancy and multiple pregnancies (3). Goldman etc. conducted a RCT in women ≥38 years with unexplained infertility to evaluate therapy begun with IUI/OS *vs*. immediate IVF. They discovered that after two cycles of treatment, IVF was associated with a greater CLBR (4).

Reichman et al. compared the effectiveness of IVF *versus* IUI for patients with poor ovarian response (defined as ≤3 follicles ≥14mm on the day of hCG administration). They found that IVF was superior to IUI in pregnancy rate when there were ≥2 follicles (5). Quinquin et al. also concluded that for poor responders defined by Bologna criteria, live birth rate was higher in the IVF groups compared with that in the IUI groups in the setting of two follicles ≥16 mm on the day of hCG triggering (6). Elzeiny evaluated the effects of IVF and IUI in couples with unexplained infertility when only 2-3 mature follicles were stimulated by the same dosage of gonadotropin. Their study demonstrated that the IVF group had a greater live birth rate and lower cost per live birth (7).

The previous studies’ findings are unclear, thus whether IVF is preferable to IUI for the treatment of unexplained infertility remains debatable. Although some research focused on effectiveness of IVF *versus* IUI for poor-responders. But actually poor response is not equal to low ovarian reserve. Furthermore, these trials were done at a time when IVF live birth rates were substantially lower than they are now. Due to various advancements in ovarian stimulation and embryo culture technologies during the last two decades, IVF live birth rates have improved considerably (8, 9). In comparison, IUI livebirth rates have been constant throughout the last few decades. Furthermore, no studies have been done to examine the efficacy of IVF and IUI on young patients with unexplained infertility who have an inadequate ovarian reserve, although this is becoming an increasingly significant indication for IVF (7).

As a result, the current study sought to compare the efficacy of IVF/ICSI with IUI in Poseidon group 3 patients with unexplained infertility (PG3&UI).

## Materials and Methods

### Methods

This was a retrospective analysis of PG3&UI patients who had IVF/ICSI or IUI at Third Affiliated Hospital of Guangzhou Medical University between January 1, 2015, and March 31, 2021. The study’s goal was to assess the efficacy of IVF/ICSI and IUI for the treatment of PG3&UI patients.

### Participants

All female Poseidon group 3 patients who had IVF/ICSI or IUI in the Department of Reproductive Medicine, the Third Affiliated Hospital of Guangzhou Medical University, were screened. The following were the inclusion criteria: (1) Poseidon group 3, i.e., female age < 35 years and AMH<1.2 ng/ml; (2) Unexplained infertility, which was defined as a menstrual cycle of 21-35 days; bilateral tubes patent confirmed by the hysterosalpingogram or laparoscopy; normal sperm analysis (concentration ≥15 million per mL, progressive motility ≥32% and morphologically normal sperms ≥4.0%) (10) and normal sexual function. (3) Only the first oocyte retrieval (OR) cycle with the first embryo transfer (ET) cycle (if there were embryos available for transfer) was included in the research for IVF/ICSI patients. For IUI groups, all eligible patients’ cycles were included in the analysis.

**Figure 1:**
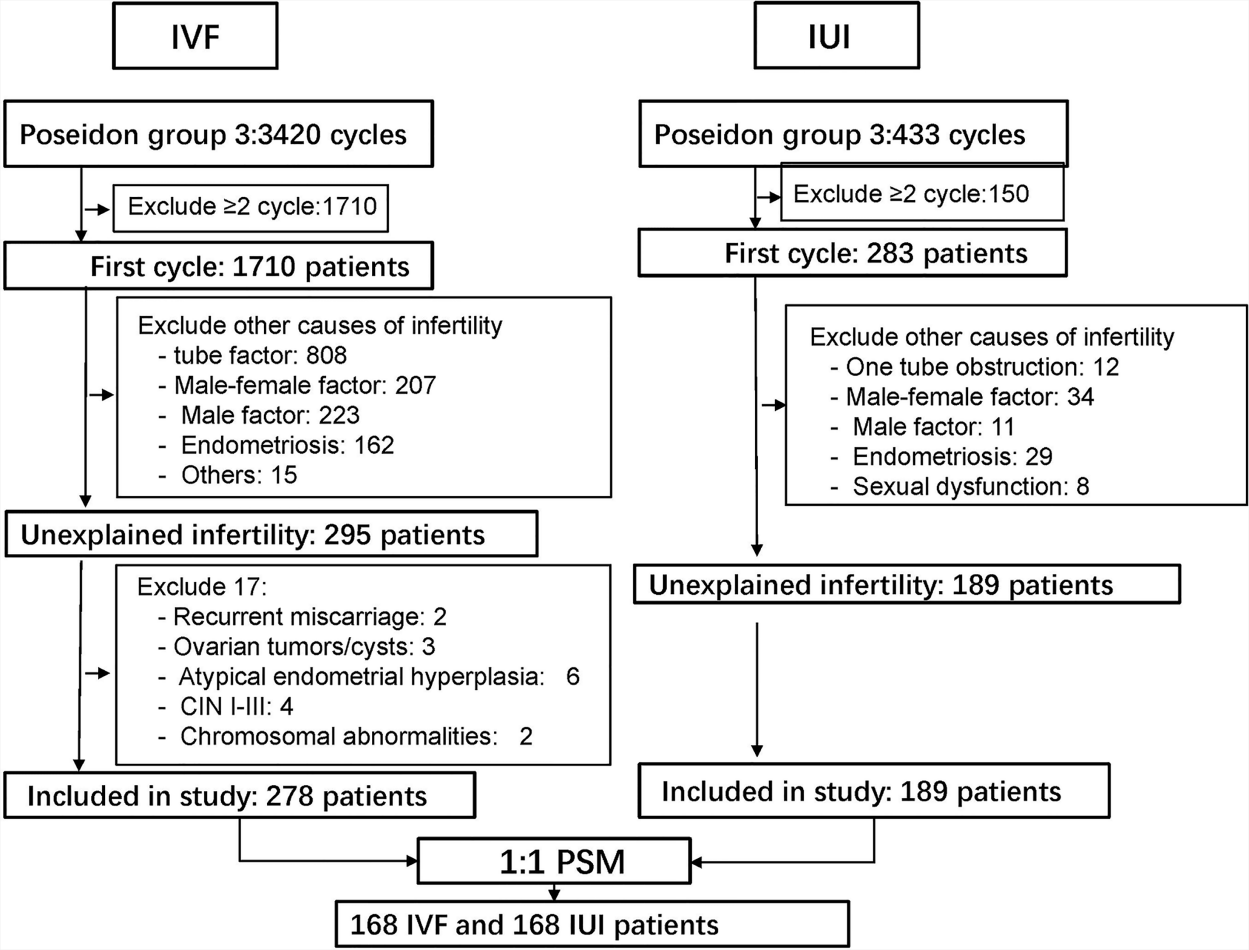
Process of screening patients.

The exclusion criteria included (1) recurrent miscarriage; (2) ovarian tumors/cysts; (3) atypical endometrial hyperplasia; (4) cervical intraepithelial neoplasm (CIN) I-III; (5) chromosomal abnormalities; (6) intrauterine adhesion and (7) cycles with donor sperm. The detail of patient screening was showed in (**Figure 1**).

### Treatment Protocol

For ovarian stimulation in IVF/ICSI cycles, mainly three protocols were used: mild stimulation, agonist, and antagonist. On cycle days 2-3, patients began mild ovarian stimulation with FSH 150 IU/day and clomiphene citrate (CC, 100–150 mg/day). When a 14-mm diameter leading follicle appeared, an antagonist was employed to inhibit the luteinized hormone (LH) surge. The antagonist protocol was similar to the mild stimulation protocol, except that the ovarian stimulation dosage was 150-300 IU of FSH (Gonal-F, Merck Serono, S.p.A.) without clomiphene citrate at the start. For the agonist protocol, triptorelin acetate 1.0 mg was given in the mid-luteal phase of the previous cycle, followed by 150–300 IU of FSH (Gonal-F, Merck Serono, S.p.A.) commencing 14 days following downregulation. When there were three 17-mm lead follicles, 250 μg of recombinant hCG (Ovitrelle, Merck Serono, S.p.A.) or 5000-10000 IU urinary hCG (HCG, Livzlon, China) was administered subcutaneously. The oocytes were retrieved 36 h later, and the embryos were transferred 3-5 days later. All embryos were frozen for the CC cycles because the endometrial thickness was decreased (11), and embryo transfer was done in another cycle, either natural or artificial. For luteal-phase support, 90 mg vaginal progesterone (Crinone, Merck Serono, England) was administered once daily three (for cleavage-stage embryo) or five days (for blastocyst) before embryo transfer and continued until the 10^th^ week of gestation if pregnancy was achieved.

Natural or stimulation protocols were employed for IUI. CC (50 mg/day), letrozole (5mg/day), or gonadotropin (37.5-75 IU/day) was started on cycle days 2-4 for cycles for ovarian stimulation. On cycle days 10-12, ultrasound and blood hormone assays (including estradiol, LH, and progesterone) were conducted, and further monitoring was scheduled based on follicle size. When the lead follicle reached 18 mm, 5000-10000 IU urinary hCG or 250 μg of recombinant hCG (Ovitrelle, Merck Serono,S.p.A.) was given, followed by IUI 36-h later. IUI was conducted 24 h after the LH rise in the cycle. Dydrogesterone (10 mg twice per day) was used to support the luteal phase during the ovarian stimulation cycle.

The serum β-hCG test was done 14 days following the ET or IUI, and luteal-phase support was continued until the tenth week in the case of an intrauterine pregnancy.

### Outcomes Measures

The primary outcome was the number of live births per couple. The IVF/ICSI group contained only live births from one oocyte retrieval with at most one ET, whereas the IUI group included live births from all eligible patients’ cycles. Clinical pregnancy, early miscarriage, and ectopic pregnancy, time to biochemical pregnancy leading to live delivery, cost, and time spent per live birth were secondary outcomes.

### Definitions

An intrauterine/extrauterine gestational sac identified by ultrasonography with positive serum β-hCG was considered as clinical pregnancy. Early miscarriage was defined as fetal growth stop or the absence of heart activity in the gestational sac within the first 12 weeks of pregnancy. The term “ongoing pregnancy” denoted a pregnancy that was more than 12-week-old and had heart activity. Live birth is defined as a pregnancy that continues with a live fetus after 28 weeks of gestation. Time to biochemical pregnancy leading to live birth defined as the time between enrollment (when the patients registered for IVF/ICSI/IUI) and positive serum hCG test (usually 14 days after ET or IUI).

### Cost-Effectiveness Analysis

Medical and non-medical expenses are included in the expenditures. The medical costs for IVF/ICSI included the enrollment examination, medications for ovarian stimulation, follicle monitoring, oocyte retrieval, sperm processing, embryo culture and transfer, embryo freezing and thawing in frozen-thawed ET cycles, and medication for luteal phase support until the pregnancy test. The cost of IUI includes the enrollment exam, medications for ovarian stimulation, follicle monitoring, sperm processing, IUI, and medication for luteal phase support till the pregnancy test. Non-medical expenditures included transportation, lodging, and time away from work. The total expenses per live birth were used to calculate cost-effectiveness.

### Statistical Analysis

Since this was retrospective research, the baseline characteristics of the IVF/ICSI and IUI groups differed. As a result, PSM was employed to screen a group of patients so that the baseline characteristics of the two groups were identical. The propensity score was calculated using the multiple logistic regression models, with IVF/ICSI *vs*. IUI as the dependent variable and female age, duration of infertility, AMH level, and BMI as independent factors. The PSM was carried out using a caliper width of 0.2 of the standard deviation (SD) of the logit of the propensity score and 1:1 matching by closest neighbor matching. SD for baseline variables before and after PSM was computed; an absolute value less than 0.1 indicated a minor imbalance.

To compare live birth rates, we ran the following analyses: ITT analysis of all patients in the two groups following PSM (one oocyte retrieval cycle with at most one ET *vs*. all IUI cycles); PP analysis excluding women who cancelled the oocyte retrieval or IUI; *post-hoc* sensitivity analysis comparing the effectiveness of two IUI cycles *vs*. one oocyte retrieval cycle with at most one ET, excluding 81 IUI patients performing ≤1 cycle without live birth. Only the first two cycles of IUI were analyzed for individuals who had ≥3 cycles of IUI. We also performed a *post-hoc* sensitivity analysis to evaluate the efficacy of 2 IUI cycles *vs*. one ET cycle, with only patients who had ET included in the IVF/ICSI group.

SPSS version 22.0 software was used for statistical analysis (IBM, Armonk, NY, USA). The means of quantitative variables with homogeneous variance were compared using the Student’s t-test. Quantitative variables with heterogeneous variance were represented as the median (1st and 3rd quartiles), and the Mann-Whitney U test was used to compare the medians. Risk ratios (RR) with 95% confidence intervals (CI) were computed for dichotomous variables, and the χ² test was used to determine significance. A log-rank (mantel-Cox) test was used to evaluate the time to biochemical pregnancy leading to live birth between the IUI and IVF/ICSI groups using Kaplan–Meier curves. *P* < 0.05 was deemed statistically significant.

### Ethical Approval

The Ethics Committee at the Third Affiliated Hospital of Guangzhou Medical University authorized the study [Ethic no. (2021) 096].

## Results

### Baseline Characteristics of Patients Before and After PSM

After screening, 278 IVF/ICSI and 189 IUI participants were enrolled in the research. However, after PSM, there were only 168 patients in each group. Before PSM, the duration of infertility and AMH were substantially different (|SD| >0.1) between the two groups. Female age, infertility duration, AMH, and BMI were all balanced between the two groups after matching. In the IVF/ICSI and IUI groups, the female age was 31.0 ± 2.5 years and 30.9 ± 2.4 years, respectively (|SD| <0.1). The IVF/ICSI group had an AMH level of 0.82 ± 0.28 ng/mL, which was similar to the IUI group (0.83 ± 0.27 ng/mL) (|SD| <0.1) (**Table 1**).

**Table 1:** Characteristics of patients before and after PSM.

Characteristics	Before matching			After matching		
	IVF group	IUI group	Standardized difference	IVF group	IUI group	Standardized difference
N	278	189		168	168	
Female age (year)	31.0 ± 2.6	30.7 ± 2.4	0.095	31.0 ± 2.5	30.9 ± 2.4	-0.005
infertility duration (year)	3.94 ± 2.2	3.52 ± 2.13	0.190	3.72 ± 1.93	3.64 ± 2.20	0.034
AMH (ng/mL)	0.60 ± 0.33	0.86 ± 0.27	-0.533	0.82 ± 0.28	0.83 ± 0.27	-0.013
BMI (kg/m^2^)	21.3 ± 3.3	21.5 ± 3.1	-0.067	21.4 ± 3.1	21.3 ± 2.8	0.024
No. of previous deliveries	0.10 ± 0.35	0.13 ± 0.35	NA	0.13 ± 0.37	0.13 ± 0.36	NA
Male age (year)	32.8 ± 3.5	32.6 ± 3.9	NA	32.9 ± 3.6	32.8 ± 3.8	NA
AFC	6.4 ± 2.6	7.9 ± 2.3	NA	7.0 ± 2.6	7.7 ± 2.2	NA
Baseline FSH (IU/L)	8.39 ± 4.65	6.84 ± 2.63	NA	7.65 ± 4.10	6.86 ± 2.64	NA

PSM for female age, infertility duration, AMH and BMI. NA, not applicable.

### Characteristics and Outcomes of IVF/ICSI and IUI

**Figure 2:**
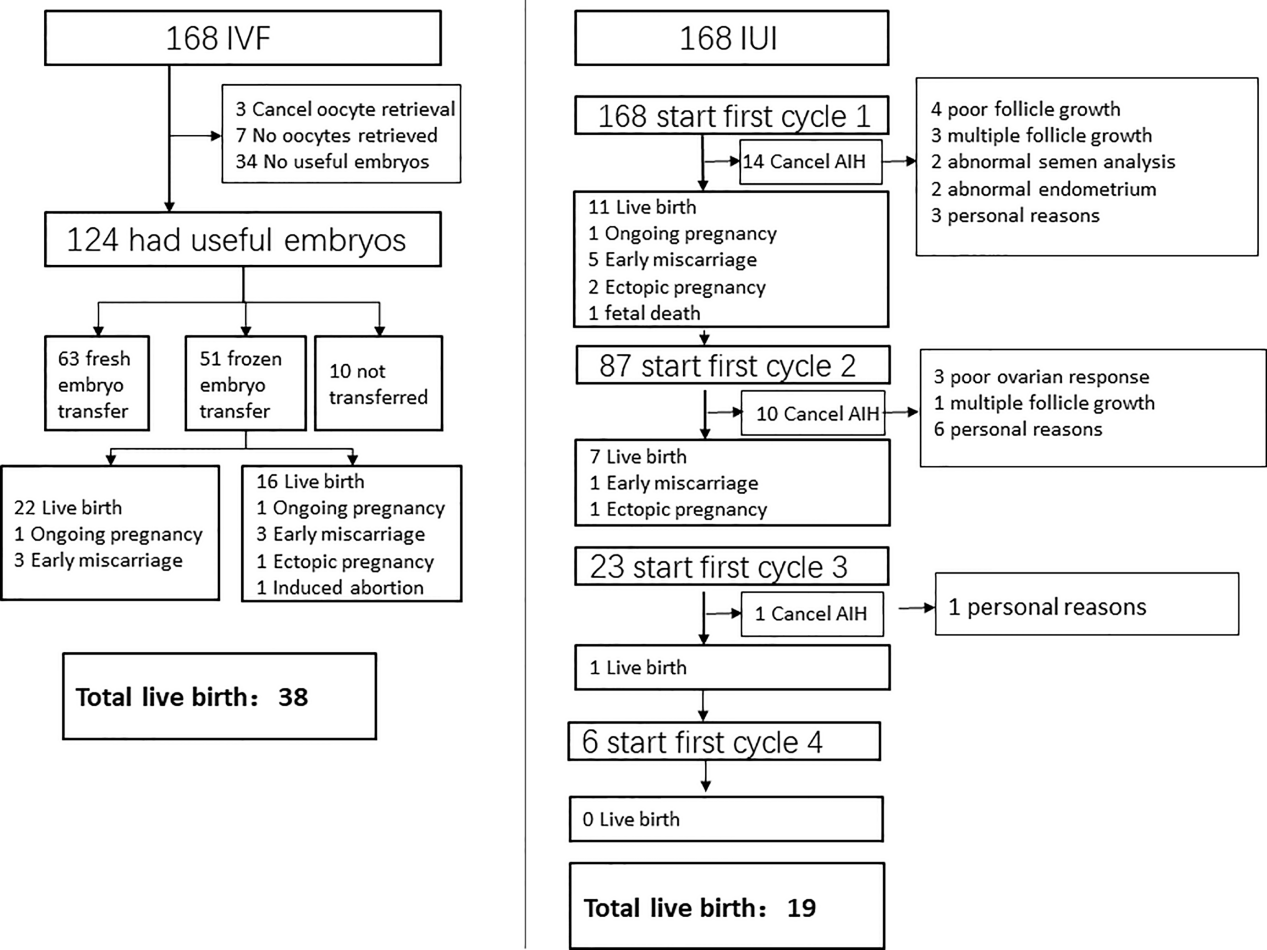
Outcomes of patients following IVF and IUI.

Characteristics of IVF/ICSI and AIH cycles were shown in **Tables 2**, **3**. Three of the 168 patients who began IVF/ICSI had their oocyte retrieval terminated owing to poor ovarian response, 7 (4.2%) had no oocytes retrieved, and 34 (20.2%) had no transferable embryos. There were 63 fresh embryo transfers and 51 frozen embryo transfers. The frozen embryos from the first cycle had not been transferred to the remaining 10 patients since they had begun another IVF/ICSI cycle and had live births. The 114 embryo transfer cycles resulted in 38 live births (22.6% per OR cycle and 33.3% each ET cycle), two ongoing pregnancies (beyond 12 weeks currently), six miscarriages, one ectopic pregnancy, and one induced abortion (due to major birth defect) (**Figure 2** and **Table 4**). Fourteen of the 168 patients who started IUI had their first IUI cycle terminated due to inadequate follicle growth, multiple follicle growth, or abnormal semen analysis, etc. (**Figure 2**). The remaining 154 IUI cycles resulted in 11 live births, 5 miscarriages, one onging and one ectopic pregnancy, and one fetal death. There were 87 patients starting cycle 2, with 10 having IUIs cancelled for various reasons. The 77 IUI cycles resulted in seven live births, one miscarriage, and one ectopic pregnancy. Only 23 patients began cycle 3 and 6 began cycle 4, resulting in only one live delivery (**Figure 2** and **Table 4**). In all, 19 (11.3%) live births occurred in the IUI group, compared to 38 (22.6%) live births in the IVF/ICSI group.

**Table 2:** Ovarian stimulation and embryo transfer of the IVF/ICSI cycles.

Parameters	IVF/ICSI group
n	168^a^
Stimulation protocols % (n)	
Mild stimulation	43.5 (73)
Agonist protocol	22.6 (38)
Antagonist protocol	16.7 (28)
Others	17.3 (29)
Starting dose of Gn (IU)	186 ± 62.6
Days of Gn	9.5 ± 3.1
Total dose of Gn (IU)	1977 ± 1144
No. of oocyts retrieved	4.3 ± 3.1
No. of transferrable embryos	1.64 ± 1.55
Embryo transfer %(n)	
Fresh	37.5 (63)
Frozen	30.4 (51)
Not transferred	6.0 (10)
No useful embryos	26.2 (44)
No. of embryos transferred %(n)	
1	42.1 (48)
2	57.9 (66)
Pregnancy rate per transfer cycle %(n)	
Fresh	41.3 (26/63)
Frozen	43.1 (22/51)
Ongoing pregnancy rate per transfer cycle %(n)	
Fresh	36.5 (23/63)
Frozen	33.3 (17/51)

^a^Included all the started cycles.

**Table 3:** Cycle Characteristics of the AIH cycles.

Parameters	AIH group
n	259^a^
Stimulation protocols %(n)	
Natural cycle	35.5 (92/259)
Stimulation cycle	64.5 (167/259)
Gn	74.8 (125/167)
CC	18.0 (30/167)
LE	7.2 (12/167)
No. of follicles ≥14mm on trigger day	1.2 ± 0.5
Endometrium thickness on trigger day (mm)	9.3 ± 2.2
Sperm with progressive motility after processing (%)	89.9 ± 10.7
No. of sperm with progressive motility after processing (million/ml)	30.5 ± 23.6
Time of insemination	
Before ovulation	74.9 (194/259)
After ovulation	20.8 (54/249)
Not follow up	4.3 (11/249)
Pregnancy rate per insemination cycle %(n)	11.58 (30/259)
Ongoing pregnancy rate per insemination cycle %(n)	8.11 (21/259)

^a^Exclude the cancelled IUI cycles.

**Table 4:** Pregnancy outcomes of the IUI and IVF/ICSI patients after PSM.

	IVF group (n = 168)			IUI group (n = 168)					RR (95% CI)	*P*
	Fresh ET	Frozen ET	Total	Cycle 1	Cycle 2	Cycle 3	Cycle 4	Total		
No. of cycles	63	51	114	168	87	23	6	284		
No. of IUI cycles^a^				154	77	22	6	259		
Livebirth	22	16	38	11	7	1	0	19	2.00 (1.204-3.32)	0.006
Ongoing pregnancy	1	1	2	1	0	0	0	1	NA	NA
Clinical pregnancy	26	22	48	20	9	1	0	30	1.60 (1.07-2.39)	0.020
Miscarriage	3	3	6	5	1	0	0	6	1.00 (0.33-3.04)	1.000
Ectopic pregnancy	0	1	1	2	1	0	0	3	0.33 (0.04-3.17)	0.623^d^
Others	0	1^b^	1	1^c^	0	0	0	1	NA	NA

^a^Exclude cycles cancelled without performing IUI. ^b^Induced abortion due to major birth defects. ^c^1 still birth; ^d^Fisher exact test.

According to ITT analysis, the live birth rate for the IVF/ICSI group was substantially higher than the CLBR for the IUI group [22.6% (38/168) *vs*. 11.3% (19/168), RR 2.00, 95% CI 1.20-3.32, *P* = 0.006]. The IVF/ICSI group had a substantially higher live birth rate per cycle than the IUI group [22.6% (38/168) *vs*. 6.7% (19/284), RR 3.38, 95% CI 2.02-5.67, *P* = 8.230E-7; **Tables 4** and **5**]. In the PP analysis, which excluded 3 IVF/ICSI patients who cancelled oocyte retrieval and 6 IUI patients who canceled IUI, the livebirth rate in the IVF/ICSI group was 23.0% (38/165) and 11.7% (19/162) in the IUI group (RR = 1.96, 95% CI 1.18-3.26, *P* = 0.007). The effectiveness of one oocyte retrieval cycle and 2 IUI cycles was assessed in the *post-hoc* sensitivity analysis. Patients with ≤1 cycle of IUI without live birth were eliminated from the analysis in the IUI group. Only the CLBR of the first two cycles of IUI were computed for patients who had ≥3 cycles of IUI. The live birth rate was not substantially different between the two groups in this situation [23.0% (38/165) *vs*. 20.7% (18/87), RR 1.11, 95% CI 0.68-1.83, *P* = 0.675]. When the efficacy of one ET cycle was compared to the efficacy of two IUI cycles, one ET cycle had a higher live birth rate than two IUI cycles [33.3% (38/114) *vs*. 20.7% (18/87), RR 1.61, 95% CI 1.61 0.99-2.62, *P* = 0.048] (**Tables 4**, **5**).

**Table 5:** Livebirths by intention-to-treat and per-protocol analyses.

	IVF	IUI	RR (95% CI)	*P*
Intention-to-treat analysis	22.6 (38/168)	11.3 (19/168)	2.00 (1.20-3.32)	0.006
Intention-to-treat analysis^a^	22.6 (38/168)	6.7 (19/284)	3.38 (2.02-5.67)	8.230E-7
Per-protocol analysis^b^	23.0 (38/165)	11.7 (19/162)	1.96 (1.18-3.26)	0.007
*Post-hoc* per-protocol analysis^c^	23.0 (38/165)	20.7 (18/87)	1.11 (0.68-1.83)	0.675
*Post-hoc* per-protocol analysis^d^	33.3 (38/114)	20.7 (18/87)	1.61 (0.99-2.62)	0.048

^a^One started cycle of IVF vs. one started cycle of IUI. ^b^Excluded 6 cycles in the IUI group (cancelled IUI) and 3 cycles in the IVF groups (cancelled the oocyte retrieval). ^c^Two IUI cycles vs. one oocyte retrieval cycle (Excluded 81 IUI cycles that had ≤1 cycle without live birth. For IUI patients with ≥3 cycles of IUI, only the CLBR of the first two cycles were calculated. Exclude 3 IVF cycles that cancelled the oocyte retrieval). ^d^2 IUI cycles vs. 1 embryo transfer cycle (Excluded 81 IUI cycles that had ≤1 cycle and no live birth. For IUI patients with ≥3 cycles of IUI, only the cumulative live birth of the first two cycles were calculated. Only the 63 fresh ET and 51 frozen ET were included in the IVF groups).

### Time to Biochemical Pregnancy Leading to Live Birth

**Figure 3:**
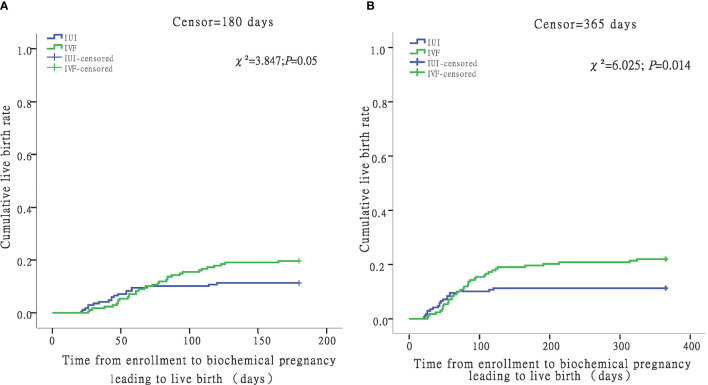
Time from enrollment to biochemical pregnancy leading to live birth in the IUI and IVF groups. For women with livebirth, time to biochemical pregnancy leading to live birth was defined as the number of days between enrollment and the date of hCG test. Women without livebirth were censored at 180 days **(A)** and 365 days **(B)**.

When censored at 365 days, the Kaplan-Meier analysis revealed that the IVF/ICSI group had a higher live birth rate than the IUI group (log-rank test χ²= 6.025; *P* = 0.014) (**Figure 3B**). However, when the two groups were censored at 180 days, the live birth rates were not substantially different (log-rank test χ²= 3.847; *P* = 0.05) (**Figure 3A**). In all, 1612 hospital visits were made in the IUI group while 1836 hospital visits were made in the IVF/ICSI group. The number of hospital visits per live birth in the IUI group was higher than in the IVF/ICSI group (85 *vs*. 48) (**Table 6**).

**Table 6:** No. of hospital visits for IVF and IUI groups.

Type of cycles	Total hospital visits	livebirths	Hospital visits/livebirth
AIH total hospital visits	1612	19	85
Female	1209		
Male	403**^a^**		
IVF total hospital visits	1836**^b^**	38	48
Female	1377		
Male	459**^a^**		

^a^hospital visits of the male were calculated as 1/3 of the female. ^b^Ten patients in the IVF groups weren’t calculated in the time spending, because they had not any embryo transfer though they had frozen embryos.

### Costs Per Live Birth for IVF/ICSI and IUI

The overall cost of live birth was comparable across the two groups (¥132242 *vs*. ¥131611), while the medical expenses for a live delivery from IVF/ICSI were higher than those from IUI (¥118955 *vs*. ¥108279). When compared to the IUI group, the expenditures per couple for IVF/ICSI were substantially greater (¥31805 *vs*. ¥14885) (**Table 7**).

**Table 7:** Cost-effectiveness analysis.

Type of cycles	Total cost (¥)	Livebirths	Cost/livebirth (¥)	couples	Cost/couple (¥)
IUI total costs^a^	2500608	19	131611	168	14885
Medical costs	2057308		108279		12246
Non-medical costs^b^	443300		23332		2639
IVF total costs	5025209	38	132242	158^c^	31805
Medical costs	4520309		118955		28610
Non-medical costs	504900		13287		3196

^a^total costs=medical costs+ non-medical costs. ^b^Indirect costs=costs of transportation, lodging, and time away from work. ^c^Ten patients in the IVF groups weren’t calculated in the cost. The frozen embryos from the first cycle had not been transferred since they had begun another IVF cycle and had live births.

### Neonatal Outcomes After IVF/ICSI and IUI

Six twins were born in the IVF/ICSI group, while all live births in the IUI group were singletons. The infant outcomes were comparable between the two groups, including birth weight, height, gestational days, preterm delivery, and low birth weight. There was one birth defect in the IVF/ICSI group and one still birth in the IUI group (**Table 8**).

**Table 8:** Neonatal outcomes after IVF and AIH.

Newborn outcomes	IVF	IUI	*P*
	38	19	
Singleton %(n)	84.6 (32/38)	100 (19/19)	0.164**^a^**
Twin %(n)	15.4 (6/38)	0	
Birth weight (g)	2956 ± 676	2886 ± 811	0.537
Birth height (cm)	47.5 ± 8.1	49.2 ± 2.1	0.356
Gestational days (day)	268 ± 19	271 ± 16	0.490
Gestational weeks<37%(n)	15.8 (6/38)	26.3 (5/19)	0.478**^a^**
Gestational weeks<32%(n)	7.9 (3/38)	5.3 (1/19)	1.000**^a^**
Birth weight<2500g %(n)	15.8 (6/38)	21.1 (4/19)	0.717**^a^**
Birth weight<1500g %(n)	2.6 (1/38)	0	1.000**^a^**
Fetal death %(n)	0	5.3 (1/19)	0.333**^a^**
Birth defect %(n)	5.3 (1/38)	0	1.000**^a^**

^a^Fisher exact test was used.

## Discussions

Expectant management, IUI/OS, and IVF/ICSI are the three therapeutic options for unexplained infertility. Farquhar et al. demonstrated that three cycles of IUI/OS outperformed three cycles of expectant care in CLBR for unexplained infertility (12). However, the efficacy of IVF/ICSI as compared to IUI is highly debated. When evaluating treatment options, five factors must be considered: The first is effectiveness (i.e., live birth rate or CLBR); the second is cost (including medical and non-medical costs); the third is time spending (time to live birth and time spent on treatment); the fourth is the patients’ physical and psychological burden; and the last is maternal, fetal, and neonatal safety (13).

Several studies have been conducted to compare the efficacy of IVF/ICSI with IUI. Angelique and her colleagues compared six cycles of IVF/ICSI against six cycles of IUI and six cycles of IUI/OS. They observed that, whereas the IVF/ICSI group had a higher live birth rate each cycle (12.2% *vs*. 7.4% and 8.7%, respectively; *P* = 0.09), the total live birth rates were comparable across the three groups. The dropout rate in the IVF/ICSI group, on the other hand, was as high as 45% which would have an impact on the efficacy of IVF (2). Three more studies (3, 14, 15) compared the efficacy of one cycle of IVF to three cycles of IUI/OS and discovered no benefit to IVF. However, the first two studies (3, 15) failed to determine the sample size required to detect the difference in the primary outcome. The sample size in each group was only 58 and may be too small to make the difference significant. Although the third study calculated the sample size and planed 125 couples for each arm, the study ended when only 207 couples were enrolled because of fund withdrawal. Therefore, these three studies were of low quality and the conclusions were not convincing enough (14).

The preceding trials were done at a time when the live birth rate of IVF was much lower (12-24.7%) than it is now (42.23% in China in 2018). The live birth rate of IUI has been pretty stable over the last two decades: 7-10% in the studies mentioned above *vs*. 10.7% in China in 2018. As a result, with the current state of Assisted Reproductive Technology (ART), it is critical to reassess the effectiveness of IVF *vs*. IUI/OS. According to the Chinese Society of Reproductive Medicine’s (CSRM) 2018 Annual Report on Assisted Reproductive Technology (ART), poor ovarian reserve (POR) accounted for 11.84% of IVF indications (9). POR patients who do not have additional infertility problems are a subset of individuals with unexplained infertility who have impaired ovarian function. Although POR has become an important indication for IVF, it is still uncertain if IVF is better than IUI for patients with unexplained infertility and POR (UIPOR). As a result, we conducted this study to compare the efficacy of IVF with IUI in patients with UIPOR.

We looked at patients in Poseidon group 3 (age<35 and AMH <1.2 ng/mL) who had no other known reasons for infertility. To equalize the baseline features of the two groups, we employed the PSM. In the ITT analysis, our study found that the live birth rate in the IVF/ICSI group was substantially higher than the CLBR in the IUI group (22.6% *vs*. 11.3%, RR 2.00, 95% CI 1.20-3.32, *P* = 0.006). However, the mean cycles in the IUI groups were just 1.69. Because this is a retrospective, real-world study, comparing the efficacy of one IVF cycle with three IUI cycles is challenging, as it would be in a well-designed RCT. Because of their low ovarian reserve, POR patients are frequently concerned about their prognosis. They would prefer IVF as their first-line treatment since they feel it has a significantly greater pregnancy rate than IUI. As a result, UIPOR patients decline IUI after 1-2 unsuccessful cycles. IVF/ICSI had a live birth rate of 22.6% per begun cycle, compared to 6.7% per initiated cycle in the IUI group. The CLBR of three cycles of IUI is thought to be equivalent to one cycle of IVF/ICSI. In our facility, individuals with unexplained infertility are advised to undergo two cycles of IUI before doing IVF. So, we compared the CLBR of two IUI cycles in 87 patients to the LBR of one IVF/ICSI cycle and discovered that the LBRs were identical in both groups: 20.7% *vs*. 23.0% (*P* = 0.675). We should be very careful about this conclusion since individuals who had only one IUI cycle were omitted from the research, and their prognosis may have been poorer.

When evaluating a treatment approach, a cost-effectiveness analysis is critical. Goverde et al. examined the CLBR for 6 cycles of IVF, 6 cycles of IUI, and 6 cycles of IUI/OS. They discovered that the IUI with natural cycle was the least expensive of the three procedures (2). Tjon-Kon-Fat et al. evaluate the expenses of three IVF-single embryo transfer (SET) cycles to six IVF- modified natural cycle (MNC) cycles *vs*. six IUI/OS cycles. When compared to IUI-controlled ovarian hyperstimulation (COH) (€5070), the mean expenses per couple for IVF-MNC (€8206) and IVF-SET (€7187) were substantially higher. When compared to IUI/OS, the cost of an extra live birth *via* IVF-SET would be €43 375 (16). Nandi et al. compared the efficacy of one IVF cycle to 3 IUI/OS cycles and determined that the expenses per livebirth for IVF were higher than for IUI, with a cost ratio of 1.3:1 (14).

All three of the preceding studies solely analyzed medical expenses, demonstrating that IVF was more expensive than IUI. They did, however, overlook non-medical expenditures such as transportation, lodging, and time away from work. When we assessed the medical expenditures per live birth in our study, the costs for IVF were ¥10 676 greater than the costs for IUI. When the overall expenses per live birth, which included non-medical charges, were assessed, the prices for IVF/ICSI and IUI were relatively similar. Our system didn’t record the expenses beyond enrollment; therefore, we only computed the total expenditures from enrollment until the time of biochemical pregnancy.

Time to live birth and hospital visits on the therapy are two indicators used to assess the amount of time spent on fertility treatment. We didn’t utilize the criterion “time to live birth” since it only included patients who had a live delivery and ignored the time spent by those who didn’t. Because of the intricacy of IVF, the time to live delivery may be longer than with IUI. To compare the time spent for the two procedures, we utilized the Kaplan-Meier curves, which is recommended for evaluating the CLBR across time in clinical investigations (17). The findings showed that when the two groups were censored at 180 days, the live birth rates were not substantially different. However, when censored at 365 days, IVF/ICSI group had a higher live birth rate than IUI group (log-rank test χ²= 6.025; *P* = 0.014). There have been no prior studies that focused on hospital visits for fertility treatment. We tallied the hospital visits and discovered that IUI patients required more hospital visits per live delivery than IVF/ICSI patients (85 *vs*. 48). The majority of patients have employment, and they frequently have to request time off for treatment, which has a detrimental impact on their productivity and income. Furthermore, repeated hospital visits exhaust and stress them. From this perspective, IUI is less patient-friendly than IVF.

The safety aspects included: complications of ovarian stimulation (e.g., ovarian hyperstimulation, OHSS), oocyte aspiration (e.g., pelvic hemorrhage), maternal and fetal problems during pregnancy (mostly multiple pregnancies and birth abnormalities), and natal complications (e.g., premature delivery). There were two occurrences of late OHSS in the IVF/ICSI group and none in the IUI group in the current research. Except for 6 twins and one birth defect in the IVF/ICSI group and 1 fetal death in the IUI group, the neonatal results were similar between the two groups.

The current study has the following advantages: first, it is the first to assess treatment options for Poseidon group 3 and unexplained infertility patients. Second, PSM was utilized to equalize the baseline features of the two groups, reducing the impact of baseline on live birth. Third, while assessing cost-effectiveness, we included non-medical expenditures, which represent the couples’ real costs. Finally, we compute the number of hospital visits per live birth, which represents the patients’ physical and psychological stress.

The drawbacks are as follows: first, because this is retrospective research, it cannot adequately evaluate the efficacy of one IVF cycle with three cycles of IUI. In our study, the average number of IUI cycles was only 1.67. However, it is real-world research that represents the patients’ and physicians’ actual alternatives. Second, while assessing cost-effectiveness, the expenditures associated with pregnancy and delivery was not considered. Because there were six twin pregnancies in the IVF/ICSI group, the expenses of IVF will be underestimated.

## Conclusions

In real-world research of Poseidon group 3 and unexplained infertility patients, the CLBR of IVF/ICSI with at most one embryo transfer is greater than the CLBR of IUI, with fewer hospital visits, comparable expenditures, and time to biochemical pregnancy leading to live delivery when censoring at 180 days. A randomized controlled trial should be carried out to assess the efficacy of IVF/ICSI and IUI for Poseidon group 3 and unexplained infertility patients.

## Data Availability Statement

The raw data supporting the conclusions of this article will be made available by the authors, without undue reservation.

## Ethics Statement

The studies involving human participants were reviewed and approved by The Ethics Committee at the Third Affiliated Hospital of Guangzhou Medical University. The patients/participants provided their written informed consent to participate in this study. Written informed consent was obtained from the individual(s) for the publication of any potentially identifiable images or data included in this article.

## Author Contributions

Conceptualization: YW, JL. Methodology: YW. Formal analysis and investigation: YW. Writing – original draft preparation: YW. Review and editing: HL. Funding acquisition: JL. Resources: HL. Supervision: JL. All authors contributed to the article and approved the submitted version.

## Funding

This study was supported by the National Key Research and Development Program of China (No.2018YFC1003803, to JL), the Scientific Research Program of The Third Affiliated Hospital of Guangzhou Medical University (No.2017Q15, to YW), National Natural Science Foundation of China (No.81801532 to HL), the Guangzhou Science and Technology Plan Project (No.202102010076 to HL), and the Medical Key Discipline of Guangzhou (2021-2023).

## Conflict of Interest

The authors declare that the research was conducted in the absence of any commercial or financial relationships that could be construed as a potential conflict of interest.

## Publisher’s Note

All claims expressed in this article are solely those of the authors and do not necessarily represent those of their affiliated organizations, or those of the publisher, the editors and the reviewers. Any product that may be evaluated in this article, or claim that may be made by its manufacturer, is not guaranteed or endorsed by the publisher.

## Abbreviations

IVF, *in vitro* fertilization; IUI, intrauterine insemination; PG3&UI, Poseidon group 3 patients with unexplained infertility; ICSI, intracytoplasmic sperm injection; PSM, propensity score matching; ITT, ntention-to-treat; PP, per-protocol; CLBR, cumulative live birth rate; ART, assisted reproductive technologies; IUI/OS, IUI with ovarian stimulation; RCT, randomized controlled trial; AMH, anti-müllerian hormone; OR, oocyte retrieval; ET, embryo transfer; CIN, cervical intraepithelial neoplasm; CC, clomiphene citrate; LH, luteinized hormone; hCG, human chorionic gonadotropin; SD, standard deviation; CI, confidence intervals; AFC, antral follicle count; FSH, follicle-stimulating hormone; SET, single embryo transfer; MNC, modified natural cycle; COH, controlled ovarian hyperstimulation.
